# The Origins of Jewish Guilt: Psychological, Theological, and Cultural Perspectives

**DOI:** 10.1080/19349637.2012.737682

**Published:** 2013-04-16

**Authors:** Simon Dein

**Affiliations:** Department of Mental Health Sciences, University College London, London, United Kingdom

**Keywords:** religion, guilt, Judaism, stereotype

## Abstract

The idea that guilt and Judaism are closely interlinked has a long historical legacy. After discussing recent work on anthropology and emotion focusing on shame and guilt, we examine three theories purporting to account for this link: psychoanalytic, theological, and guilt as a cultural stereotype particularly the notion of the Jewish mother.

What's Jewish Alzheimer's disease? It's when you forget everything but the guilt.

The idea of guilt is deeply ingrained in Jewish culture both in everyday discourse and is enshrined both literature and in humor. As Rabbi Harlan Wechsler (1990) asserted, deep in the Jewish tradition, deep in the Jewish Psyche of the Bible, is a human being who can experience guilt. More than guilt's being a problem is that it is second nature to the Jews. Molly Jong-Fast, Erica Jong's daughter (American poet), stated that “we suffer two great inheritances of the Jewish people: irritable bowel syndrome and guilt,” and deemed our quintessential Jewish way of life as “praying on a shrink's sofa” (Jong-Fast, 2005). From the literary perspective, the notion of Jewish guilt was famously popularized by Martin Roth in his classic *Pourtnoy's Complaint*—the story of Alexander Pourtnoy, a young Jewish man brought up by highly neurotic parents who is experiencing sexual guilt. The notion of the overbearing and highly critical Jewish mother has been a popular theme in American cinema.

But what is the origin of this guilt? Is it related in any way to Jewish theology, the history of the Jews, or is it a relatively recent cultural stereotype originating from the time of the emigration of Jews from Eastern Europe to the United States at the turn of the 20th century? Or is there something inherent in the Jewish psyche, as Freud argues, which predisposes Jews to guilt? There is little evidence that Jews as a religious and cultural group experience guilt to a greater extent than other groups, although epidemiological studies are lacking. The topic of Jewish guilt raises significant issues in relation to the emerging study of religion and emotion and, more specifically, how religious factors shape affect.

## THE ANTHROPOLOGY OF GUILT

The anthropology of emotion is a slowly growing field within social anthropology. For a long time, following Durkheim, anthropologists have ignored emotions, viewing them as capricious, subjective, and changeable; they were considered biological (Durkheim used “effervescence”), more the province of psychology and biology than anthropology. In the first few decades of the 20th century there was a divergence in the study of emotion: an emphasis on Freudian psychoanalysis in Europe and culture and personality theory in North America. From the 1970s, anthropologists began to focus more directly on emotions, questioning their nature (innate or cultural) and their role in social life. Most anthropologists would now agree that emotions are culturally constructed and that they become incorporated into the broader conceptual repertoire of culture and prevailing cultural values, and beliefs are infused into the meaning of named emotions. Not only do cultural factors shape emotions but emotions support and shape culture.

Biological and evolutionary evidence indicates that emotions are not infinitely malleable and that there are primary emotions that are largely precultural. Psychologist Paul Ekman (1971) showed that despite some idiosyncratic differences, the basic emotions—anger, fear, sadness, and happiness—are predominantly biological and thus, are universal, expressed and perceived in similar way across all cultures. Robert Levy (1984) noted that metaphors of emotion, for instance anger, are similar across cultures. At the other extreme, some assert that emotions are totally cultural and expressed in social relations, are embodied, and affect power relationships. For instance Lutz (1988, p. 5) asserted that emotional experience is not precultural but preeminently cultural. Rosaldo (1980) noted how the meaning of emotional words resides in the pragmatics of social life. They are felt thoughts in which the cultural habitus of power is embedded within the physical being of the relational self (Lindholm, 2005) and are the physical expression of authority.

Several questions arise in relation to guilt. Can we legitimately argue that the experience of guilt is the same across cultural groups? More specifically, can we assume that what biblical scholars identify as guilt in the Old Testament texts is the same as our contemporary emotion labeled guilt? Research in this area is plagued by difficulty; there are notorious difficulties translating emotional words from one language to another. Of note is the longstanding anthropological discussion of shame and guilt societies.

Ruth Benedict (1954) maintains that shame is a violation of cultural or social values while guilt feelings arise from violations of one's internal values. Thus, it is possible to feel ashamed of thought or behavior that no one knows about and to feel guilty about actions that gain the approval of others. Guilt and shame are similar emotions in that both involve feeling bad about one self. Guilt is generally associated with something one has done (or not done). There are differences in phenomenology. Shame necessitates awareness of self in relation to others; in guilt there is awareness of self in relation to some act. Guilt is more cognitive than shame and involves an obsession with violation (Lewis, 1971). In guilt, the self demands punishment for the violation. Shame, on the other hand, is often experienced as a feeling of being a bad, unworthy, hateful person. Both are states of being negatively valued by self and others, self-conscious emotions, because one has failed to meet standards of what is appropriate or right. They require an ability to see the self as an object of evaluation. Konstan (2003) noted that shame's status as a moral emotion has been impugned by critics, among them theologians and anthropologists, who consider it a primitive precursor to guilt: shame, the argument goes, responds to the judgments of others and is indifferent to ethical principles in themselves, whereas guilt is an inner sensibility and corresponds to the morally autonomous self of modern man.

Different cultures emphasize shame and guilt to different extents. Japan, for example, is a shame culture, while the United States is a guilt culture. Collectivistic cultures emphasize the fundamental relatedness of individuals to each other, for example by valuing attending to others, fitting in, and harmonious interdependence with them. In contrast, individuals in individualistic cultures hold an independent view of the self and seek to maintain their independence from others by attending to the self and by discovering and expressing their unique inner attributes. Emotion research is individualistic and American in orientation.

Cross-cultural studies suggest that the valuation, elicitors, and behavioral consequences, as well as the distinction between guilt and shame, vary across individualistic and collective cultures (Wong & Tsai, 2007). Non-Western cultures are collectivist—other people's thoughts are important and influence the self. In such cultures feeling bad about the self is valued and leads to self-improvement. In some cultural groups, distinctions between shame and guilt are blurred and may be less marked in cultures that promote an interdependent self (Wong & Tsai, 2007). Shame in many non-Western cultures is valued. Stephen Pattison (2000, p. 129) remarked, “While guilt may have a very constructive role in creating and maintaining social relationships and moral responsibilities, shame has a much more dubious effect.” Whereas Western models of shame and guilt view guilt as good and shame as bad, cross-cultural studies suggest that shame may have better and more adaptive consequences.

## WHERE DOES JEWISH GUILT DERIVE FROM?

Rabbi Jeremy Rosen, Orthodox rabbi, author, and lecturer, best known for advocating an approach to Jewish life that is open to the benefits of modernity and tolerant of individual variations while remaining committed to *halacha* (Jewish law), speculated on the origin of Jewish guilt:

Some lay the blame at the door of Christianity and its preoccupation with original sin, the Greek dichotomy between body and mind, so that body is bad, sex a concession, celibacy the ideal. This explains their traditions of self-flagellation and monastic asceticism. Perhaps it was a Medieval Jewish response to Christian Crusader piety? But that is too easy. You can find similar ideas in Jewish sources of two thousand years ago …. The Holocaust exacerbated things of course. Guilt is even stronger amongst the children of Holocaust survivors than survivors themselves. In Israel so many have lost a relative, a friend or suffered in some way. Perhaps it is the guilt of survival that weighs heavily. Or perhaps it's the realization that the wonderful dreams and ideals of Zionism, of an ethical, just society, have been lost, and we are all to blame for our current greed and corruption. (Rosen, 2008, paras. 5, 7)

These assertions have little historical backing although the description of Holocaust survivor guilt is well recognized. We shall now examine three hypotheses attempting to explain Jewish guilt: psychoanalytic, theological and religious, and the concept of the Jewish mother and assimilation. We begin with Freud's psychoanalytic theories.

## FREUD AND PSYCHOANALYSIS

Freud observed that guilt plays a fundamental role in the psyche, and that it mainly works unconsciously as the main force in the psychic causality that leads to drive renunciation and towards the development of intellectuality. Guilt, in Freud's view, derived from a violation of a law, resulting in a sense of guilt. Freud appealed to an anthropological theory, which speculates that early in civilization there was a murder of the primal father. This murder, according to Freud, is the missing link that explains the functioning of prohibition in the economy of the drives. It is the father's death that initiates the law and therefore functions as the origin of all father religions.

In *Moses and Monotheism*, Freud (1939/1967) contradicted the biblical story of Moses asserting that Moses only led his close followers into freedom during an unstable period in Egyptian history after Akhenaten. They subsequently killed Moses in rebellion later joining with another monotheistic tribe in Midian who worshiped a volcanic God. Freud argued that many years after the murder of Moses, the rebels expressed regret at their action and subsequently developed the concept of the Messiah as a hope for the return of Moses as the savior of the Israelites. According to Freud, the guilt from the murder of Moses is inherited through the generations; this guilt then drives the Jews to religion to assuage their emotions. The book consists of three parts, and like *Totem and Taboo*, is an extension of Freud's work on psychoanalytic theory as a means of generating hypotheses about historical events. As in *Totem and Taboo*, he equates religion and neurosis:

That conviction I acquired a quarter of a century ago, when I wrote my book on *Totem and Taboo* (in 1912), and it has only become stronger since. From then on I have never doubted that religious phenomena are to be understood only on the model of the neurotic symptoms of the individual, which are so familiar to us, as a return to of long-forgotten important happenings in the primeval history of the human family, that they owe their obsessive character to that very origin and therefore derive their effect on mankind from the historical truth they contain. (Freud, 1939/1967, p. 89)

*Moses and Monotheism* has been vilified and dismissed by critics on account of his “scandalous” hypothesis that claims not only that Moses was not a Jew but also that he was murdered by his own people in the wilderness. As an historical hypothesis it lacks evidence. But as philosopher Richard Bernstein (1998) noted, the book is not without merit in terms of furthering our understanding of the unconscious dynamics of religion.

## RELIGION AND GUILT

Guilt and religion have a longstanding association in Western Culture, and some, such as Belgum (1963), asserted that guilt is the place where religion and psychology meet. There are important differences in how religions use guilt as a spiritual vehicle and as a form of social control. Whereas psychology is interested in guilt as a subjective phenomenon, religions focus on guilt as moral culpability based on objective behaviors. Psychology is interested in how people behave—descriptive whereas religions focus upon how people ought to behave—prescriptive.

There is evidence that there has been a diminution in feelings of guilt in the past 100 years due to the decline in the importance of religion in Western societies (Orelli, 1954). Recent studies suggest that guilt feelings appear fairly universally across cultures, however they are more prevalent in the western world and hypochondriacal ideas are the core features of depression in non-Christian cultures. From the European Middle Ages onwards, a process of steadily increasing individualization took place, which found its culmination in the beginning of the 19th century. This process was closely linked to the transformation of a shame culture into a guilt culture. The gradual elaboration of differentiated concepts of sin, guilt, remorse, and penitence in this process was of crucial importance.

Albertsen, O'Connor, and Berry (2006) provided an excellent overview of the relationship between religion and interpersonal guilt, and the discussion here derives from them. Although religion has been found to be strongly related to a variety of psychological and health variables (Koenig, McCullough, & Larson, 2001), few studies have incorporated religion in the examination of cross-cultural differences in predisposition to guilt. It has sometimes been suggested that religion fosters guilt in people (Ellis, 1980), and some authors recognize a maladaptive, scrupulous, or penitent personality (Ciarrocchi, 1995; Spero, 1980; Van Ornum, 1997) associated with excessive worry about sin and guilt. There is anecdotal evidence that members of fundamentalist religions tend to have high levels of religious guilt and fear (e.g., Barr, 1980; Hartz & Everett, 1989; Strozier, 1994).

Although a few empirical studies have examined the relationship between guilt and religious participation, the specific type of guilt is rarely clearly defined (Albertsen et al., 2006). Following a review of over 200 studies, Gartner, Larson, and Allen (1991) asserted that low levels of religiosity are associated with impulse control disorders, including drug and alcohol use, suicide, and antisocial behavior, whereas high levels of religiosity are more often associated with disorders of over control, such as excessive guilt. Studies have reported positive correlations between religiosity and general guilt (Luyten, Corveleyn, & Fontaine, 1998), religiosity and guilt related to sexual, hostile, or immoral impulses (Fehr & Stamps, 1979), and religiosity and adaptive, shame-free guilt (Albertsen, 2002). However, to date, research has not demonstrated a direct relationship between religiosity and maladaptive interpersonal guilt (Albertsen, 2002).

Only a few studies have explored guilt across religious traditions, with individuals from the Catholic religious tradition typically experiencing higher levels of guilt. London, Schulman, and Black (1964) reported a higher guilt in Protestant and Catholic samples compared with a Jewish sample in the Midwest region of the United States. In a Dutch sample, feelings of guilt were more prevalent in Roman Catholics than in Calvinists or nonchurch members (Braam, Sonnenberg, Beekman, Deeg, &Van-Tilburg, 2000). Of note, however, is the fact that the setting of Braam's study in the Netherlands may reduce generalizability to people in the United States. In the only published study comparing religious traditions specifically on maladaptive interpersonal guilt, Catholics and Lutherans were significantly higher than Buddhists and Episcopalians in the United States (Albertsen, 2002). Such studies suggest that certain religious traditions, such as Catholicism, may tend to foster higher levels of guilt among their members, or that different religious traditions attract members—on either the community or the individual scale—with different levels of guilt. There is nothing in the tenets of Judaism that engenders guilt to a higher degree than found in most religions. This means Judaism is not any more subject to neuroticism than any other major world faith or ideology.

## GUILT AND SHAME IN THE OLD TESTAMENT

It was William Robertson Smith (1889), a Scottish orientalist, Old Testament scholar, professor of divinity, and minister of the Free Church of Scotland, who first presented a somewhat speculative account of the origins of sin and guilt in ancient Judaism following the Babylonian conquest. According to Smith, the large and powerful kingdom was divided and begin to fall: Israel first, to the Assyrians. The power of Babylon rose, Judah was besieged, and Jerusalem taken and the Temple destroyed. The people experienced adversity, downfall, and exile; the prophets criticized them for their backsliding, their whoring, their foreign women, their altars to other gods, and their luxury. The sense of joy and prosperity celebrated previously went from worship to be replaced increasingly by a sense of guilt, offense against God, and the need to pacify his just anger by expiation and propitiation. After the destruction of the kingdoms and experience of exile, the themes of sin and punishment—of the need to atone—came to dominate the whole sacrificial system, altering its character so that its focus is on sacrifice because of sin.

An understanding of guilt in Old Testament texts raises significant problems of translation. To what extent do biblical terms refer to the contemporary emotion of guilt? And were the biblical writers referring to guilt or shame? Guilt, sin, and sacrifice run closely together in the Hebrew Bible. The Hebrew Bible does not deploy a unique word for guilt, but uses a single word to signify: “sin, the guilt of it, the punishment due unto it, and a sacrifice for it.” Guilt and sin are referred to as *awon*—to bear iniquity. The term connotes lawlessness and rebellion.

The key concept underlying guilt in monotheistic religion is sin—a word derived from the Latin word *sont* meaning guilt—but the two are not synonymous. Sin is the consequence of violating a religious ordinance, whereas guilt is not a moral violation but the result of one, both as culpability and a feeling of remorse. Feelings of sin and guilt relate to God, and ideas of sin and guilt and punishment constantly pass over into each other. This is demonstrated by noting the use of the words whose common root is ‘-sh-m, the distinctive Hebrew term for guilt. In Lev. 5 to 7, in the adjective form it is rendered “guilty,” in the noun as “trespass offering.” In Hos. 5:15 it seems to mean punishment (see margin, “have borne their guilt,” and compare Ezek. 6:6), while in Nu. 5:7–8 the idea is that of compensation (rendered “restitution for guilt”). *Asham* is a guilt offering as a reparation mandated for a specific offence such as breaking an oath. It requires that the sinner make a sacrifice to God and involves paying a debt (guilt as debt). *Ashem* is translated as being guilty and *ashama* for feeling guilty.

Guilt signifies alienation from God as a result of sin and only He can absolve one from sin and guilt. All the biblical words for “sin” imply no more than an error of judgment, to miss the mark, to step off the path, to fall short. There is no “state of sin,” just mistakes that need to be avoided next time. Just get back on the path. Sin in Judaism is similar to the Greek word *hamatria*—missing the mark—making a mistake by not fulfilling the law.

Following the prophets, the ideas of sin, guilt, and righteousness developed more clearly as ethical and personal: “It is not ritual correctness that counts with God, incense and sacrifices and new moons and Sabbaths, but to cease to do evil, to learn to do well” (Isa. 1). Thus, the motive and the inner spirit come in (Mic. 6:8; Isa. 57:15; 58:1–12), with guilt gaining a new depth and quality and becoming more interiorized.

Guilt in the Old Testament is at the same time both individual and communal. The biblical word for guilt, asham, is only once used of individuals. Guilt in Judaism has a strong communal aspect—Yom Kippur is a collective petition for forgiveness. But this is also an individual act whereby the individual personally reflects and repents. *Kapporot* is a Jewish ritual practiced by some Jews on the eve of Yom Kippur. The person swings a live chicken or a bundle of coins over one's head three times, symbolically transferring one's sins to the chicken or coins—a form of guilt offering. The chicken is then slaughtered and donated to the poor for consumption at the prefast meal.

A full understanding of the relationship between guilt and sin necessitates a discussion of conscience—broadly defined as an aptitude, faculty, intuition, or judgment of the intellect that distinguishes right from wrong. In psychological terms conscience leads to feelings of remorse when a human commits actions that go against their moral values and to feelings of rectitude or integrity when actions conform to such norms.

Did the ancient Hebrews possess such a faculty? We have little direct information about conscience in the ancient Hebrews, but some understanding may be gleaned by examining surrounding cultures such as Greeks. While we have little historical evidence of direct contact between the Ancient Greeks and early Jewish culture, it is likely that such contact occurred. Dodds (1951), in *Greeks and the Irrational*, drew upon ancient Greek literature to examine the mind of the ancient Greeks. In the *Iliad*, the first clear picture of the early Greek religion, Agamemnon offers an apology for compensating himself for the loss of his mistress by stealing the mistress of Achilles. He asserts that he was not himself the cause of this act but it was due to divine intervention by Erinys, a goddess, who removed his understanding. There are numerous other passages in Homer in which unwise and unaccountable conduct is similarly attributed to supernatural agencies of one kind or another.

Dodds contended that these explanations are not instances of poetic license but are real psychological phenomena; ancient Greek psychology differed from that of contemporary Western culture. This perception influenced, among others, Julian Jaynes in his groundbreaking study *The Origin of Consciousness in the Breakdown of the Bicameral Mind*, and more recently Antonio Damasio in *The Feeling of What Happens*, two later authors who drew upon Dodds extensively.

Dodds maintained that a transition occurred from shame culture, which characterized the worldview of the Iliad, to guilt culture, which emerges in later Greek civilization. This is a central idea for which Dodds presented a persuasive case, although his account suffers somewhat from being rather strongly influenced by Freudian psychoanalytic theory, which appeared more securely founded in science in the mid-20th century than it does today.

Dodds (1951) described an increasing sophistication in their development, from a conception of the world and the moral order as arbitrary and subject to the whim of the gods, through to a later understanding of the limits of moral responsibility. Even among the great tragedians (for example, Aeschylus’ *Oresteian Trilogy*), individuals were simply caught up in the workings out of the curse of Atreus; Sophocles makes the issue of responsibility more pertinent, and for Euripides it resides more fully in the individual. Aristotle finally identified *hamartia* or “tragic fault” as an attribute of the individual.

Like many other cultures, Greece and Rome did not use distinct terms for what we now call shame and guilt, and they appear to recognize one concept where we recognize two. This view, however, presupposes a natural correspondence among psychological ideas across linguistic and social boundaries. Thus, the Greek term we customarily translate as shame is held to match, more or less, the English concept, unless perhaps, in the absence of a word for guilt, Greek shame had a somewhat wider extension so as to include some (or all) of the modern notion of guilt. Alternatively, the ancient Greeks simply failed to achieve a notion of guilt, which is in turn a sign of the poverty of their moral vocabulary and their incomplete psychological development.

There is no Hebrew term in the Old Testament that is a linguistic equivalent for the classical Greek term *suneidesis* (*suneivdhsi*), or awareness. The Hebrew term for “heart,” however, appears a prominent term of self-awareness in the Old Testament. The lack of a developed concept of conscience in the Old Testament, as is seen later in Paul, may be due to the worldview of the Hebrews. Consciousness of life was of a relationship between God and a covenant community rather than an autonomous self-awareness between a person and his or her world. The only usage of suneidesis (suneivdhsi) in the canonical section of the Septuagint is in Ecclesiastes 10:20, “Do not revile the king even in your *thoughts*, or curse the rich in your bedroom,” where it is clearly used as self-reflection in secret (cf. the only verbal variations in Job 27:6 and Lev 5:1). Rabbinic Judaism and the Dead Sea Scrolls are consistent with the Old Testament in their lacking a vocabulary of conscience.

The Old Testament does not clearly distinguish between physical and spiritual organs, because the entire range of higher human functions such as feeling, thinking, knowing, loving, keeping God's commandments, praising, and praying is attributed not only to the “spiritual” organs of the soul and spirit but also to the physical organs of the heart and, occasionally, to the kidneys and viscera. The soul (*nephesh*) and the spirit (*ruach*) in the Old Testament refer not to immaterial entities capable of surviving the body after death, but to a whole spectrum of physical and psychological functions. These terms refer not to wholly different substances, each with its own distinct functions, but to the interrelated and integrated capacities and functions of the same person. The fact that a person is comprised of various parts which are integrated, interrelated, and functionally united, undermines the notion of the soul being distinct from the body and thus removing the basis for the belief in the survival of the soul at the death of the body.

The heart in Biblical thought is seen as the spring of individual life, the ultimate source of the physical, intellectual, emotional, and volitional energies, and, consequently, the part of the person that normally has direct contact with God. The recesses of the heart contain the thoughts, the attitudes, the fears, and the hopes that determine the personality or character of the individual. The emotions of the heart are portrayed vividly and concretely. The heart is said to fail (Gen 42:28), to faint (Gen 45:26), to throb (Ps. 38:10), to tremble (1 Sam 28:5), to be stirred up (Prov. 23:17; Deut. 19:6), or to be sick (Prov. 13:12). The state of the heart dominates every manifestation of life. “A glad heart makes a cheerful countenance, but by sorrow of heart the spirit is broken” (Prov. 15:13). Health is affected by the condition of the heart. “A cheerful heart is a good medicine, but a downcast spirit dries up the bones” (Prov. 17:22). Sometimes, physical organs can refer to something similar to conscience: “David's heart smote him” (1 Sam 24:5). Kidneys have a similar meaning. “Thus my heart was grieved, and I was pricked in my reins” (Ps. 73:21).

Critics, such as Malina and Rohrbaugh (1992), asserted that scholars have confused shame and guilt, and have attributed guilt to the ancient Hebrews when they were actually referring to shame. Two instances: Psalm 38:4 is interesting, “My guilt has overwhelmed me like a burden too heavy to bear,” and Proverbs 28:17, “A man tormented by the guilt of murder will be a fugitive till death; let no one support him.”

In relation to this assertion, Rohrbaugh (2010) stated:

No, these texts do not indicate that ancient people could be overcome by guilt. They indicate that people could be overcome by shame. Understanding the difference between guilt and shame is crucial here. Guilt is an internal reaction to a violation of one's own conscience. It depends on the existence of an individual conscience—something Middle Easterners do not have.

Shame is an internalization of the moral judgment that comes from outside, from the group. In shame cultures it is the group that has the conscience, not the individual. Thus when a group accuses one of violating its standards, deep shame is the result. That is what we read about in the Bible (see 1 Cor. 4:4).

So finally, is there a relationship between Jewish practice and the development of guilt? We would argue for this possibility. Jewish exegesis involves pouring over texts and evaluating. The Hebrew word for prayer (*tefillah*) comes from the root *fallal*, to evaluate. The Hebrew root means to think, entreat, judge, or intercede; and the reflexive means to judge oneself, and to pray. People evaluate themselves. This may be conducive psychologically to the development of guilt.

## JEWISH GUILT AS A STEREOTYPE

Jewish stereotypes are commonplace in Jewish and non-Jewish culture. Common objects, phrases, and traditions used to emphasize or ridicule Jewishness include bagels, playing violin, klezmer, undergoing circumcision, haggling, and uttering phrases like “mazal tov,” “shalom,” and “oy vey.” Other Jewish stereotypes include the rabbi, the complaining and guilt-inflicting Jewish mother stereotype, the spoiled and materialistic Jewish-American princess, and the often-meek nice Jewish boy.

As Joshua Halberstam wrote in the Jewish Daily Forward (2005):

How, then, did this bromide about Jewish guilt attain its status as a distinctive Jewish disposition? Unlike jokes about *kishke* (intestines), which Jews actually ate (and eat), and such slurs such as the Jews’ association with money—originally propounded by non-Jews—the Jewish guilt syndrome is a Jewish creation, the invention of the previous generation of assimilated American Jews. When these Jews became untethered and estranged from Jewish tradition and the established forms of expiation, they created a psychologized specter of guilt as a “Jewish condition,” a Judaism so lite, it fits on an HBO laugh track and on your friend's T-shirt.

The Jewish mother or wife stereotype is one of the most common stereotypes and stock characters employed by Jewish comedians and authors whenever they discuss actual or fictional situations involving their mothers or other females in their lives who possess mother-like qualities. The stereotype comprises of a nagging, overprotective, manipulative, controlling, smothering, and overbearing mother or wife, who persists in interfering in her children's lives long after they have become adults and can care for themselves. In Israel, where the geographical background of Jews is more diverse, the same stereotypical mother is referred to as the Polish mother. Helmreich (1984) correctly noted that the attributes of a Jewish mother—overprotection, pushiness, aggression, and guilt-inducement—are found in mothers of other ethnicities, from Italians through Blacks to Puerto Ricans. The association of this otherwise gender stereotype with Jewish mothers in particular, according to Helmreich, derives from the emphasis that is traditionally placed by Judaism on the home and the family, and on the role of the mother within that family.

The Jewish mother stereotype originated among the American Jewish community, while its predecessors derived from Eastern Europe. This stereotype was further developed by the poverty and hardship of Eastern European Jews immigrating into the United States (during the period 1881–1924, when one of the largest waves of such immigration occurred), where the requirements of hard work by the parents were transmitted to children via guilt: “We work so hard so that you can be happy.” Other aspects of the stereotype originate from those immigrant Jewish parents’ ambitions for their children to be successful, resulting in a desire for perfection and a continual dissatisfaction with anything less: Hartman and Hartman (1996) speculated that the root of the stereotype is in the self-sacrifice of first-generation immigrants, unable themselves to take full advantage of American education themselves, and the consequent transference of their aspirations, for success and social status, from themselves to their children. A Jewish mother derives vicarious social status from the achievements of her children, where she is unable to achieve such status herself.

Although this stereotype was regularly portrayed in American cinema from the 1970s onwards, according to Alisa Lebow (2008), in the late 20th century and the 21st century the stereotype of the Jewish mother has all but disappeared from movies. The Jewish mother stereotype has transformed in the Jewish grandmother, or *bubbe*. While still unschooled, food-obsessed, doting, loving, anxious, and a working class *balabusta* (good home-maker), the Jewish grandmother is more mellow than her Jewish mother antecedent.

## CONCLUSION

I have previously discussed a number of perspectives on Jewish guilt: anthropological, psychoanalytic, and cultural. All provide some understanding of the relationship between Judaism and guilt. Although this article is predominantly theoretical, I end by briefly discussing the implications of Jewish guilt for psychotherapeutic work. Within the academic study of psychotherapy there has been increasing attention given to the role of cultural factors such as experiences of migration and racism (Kareem & Littlewood, 2000) and spiritual factors such as relationship with God, religious observance, and sin (Pargament, 2007) in the psychotherapeutic process. Psychological distress, according to these authors, must be understood holistically and move beyond individual biographical factors. Understanding guilt in therapy among Jews necessitates incorporating wider cultural and theological perspectives. Future work in this area should focus on the development of culturally and spiritually focused therapy for this population.
